# Researches on the Bacillus of Influenza

**Published:** 1918-11

**Authors:** 


**Researches on the Bacillus of Influenza.** *La Presse Médicale*, October 3, 1918.

MM. Charles Nicolle and Charles Lebailly report the conclusions obtained in their researches about the bacillus of influenza.

*A*) In one series of researches the authors, collecting bronchial sputum from a patient suffering for three days from influenza, injected this sputum.

1. Without filtration, to a monkey "Bonnet Chinois". This monkey was inoculated on the conjunctivae of both inferior eyelids, and by nasal instillation.

2. After filtration, to two subjects who volunteered for the experiment. One of these was injected sub-cutaneously, the other intravenously; the filtrate obtained after centrifugalizing a mixture of the bronchial secretions and normal saline, and filtration of the whole through a Chamberland filter L2, under a pressure of from 30 to 40 cm. of mercury.

The monkey suffered from high fever during three days, from marked depression, and general wasting.

The first subject suffered from headache, general pains in all limbs, a pirexial attack rising to 39° C. and all classic features of influenza. The recovery was total after 12 days.

The second subject presented no morbid symptom.

The injection of 3 cc. of blood removed from the monkey

"Bonnet Chinois" on the first day of its illness, was also followed by no morbid symptom.

*B*) The injection of a healthy subject with 3 cc. of blood removed from a patient suffering from influenza, had no bad result.

The authors consider that :

1. Bronchial secretions of patients suffering from influenza are virulent during the acute period of the disease.

2. Monkeys may be infected by inoculation through the nose and conjunctivae.

3. The specific agent of influenza seems to be some filtrating virus, since the sub-cutaneous injection of the filtrate has developed the disease in healthy subjects.

4. The virus of influenza does not seem to exist in the blood of the patients.

